# Age Effects in Postural Control Analyzed via a Principal Component Analysis of Kinematic Data and Interpreted in Relation to Predictions of the Optimal Feedback Control Theory

**DOI:** 10.3389/fnagi.2018.00022

**Published:** 2018-02-05

**Authors:** Thomas H. Haid, Aude-Clémence M. Doix, Benno M. Nigg, Peter A. Federolf

**Affiliations:** ^1^Department of Sport Science, University of Innsbruck, Innsbruck, Austria; ^2^Human Performance Laboratory, Faculty of Kinesiology, University of Calgary, Calgary, AL, Canada

**Keywords:** healthy aging, postural control, principal component analysis on kinematic data, minimum intervention principle, optimal feedback control, balance control, tandem stance

## Abstract

Optimal feedback control theory suggests that control of movement is focused on movement dimensions that are important for the task's success. The current study tested the hypotheses that age effects would emerge in the control of only specific movement components and that these components would be linked to the task relevance. Fifty healthy volunteers, 25 young and 25 older adults, performed a 80s-tandem stance while their postural movements were recorded using a standard motion capture system. The postural movements were decomposed by a principal component analysis into one-dimensional movement components, PM_k_, whose control was assessed through two variables, N_k_ and σ_k_, which characterized the tightness and the regularity of the neuro-muscular control, respectively. The older volunteers showed less tight and more irregular control in PM_2_ (N_2_: −9.2%, *p* = 0.007; σ_2_: +14.3.0%, *p* = 0.017) but tighter control in PM_8_ and PM_9_ (N_8_: +4.7%, *p* = 0.020; N_9_: +2.5%, *p* = 0.043; σ_9_: −8.8%, *p* = 0.025). These results suggest that aging effects alter the postural control system not as a whole, but emerge in specific, task relevant components. The findings of the current study thus support the hypothesis that the minimal intervention principle, as described in the context of optimal feedback control (OFC), may be relevant when assessing aging effects on postural control.

## Introduction

In the USA around 20% of people aged 65 and over are diagnosed with only fair or poor health and long-term care services alone require between 210 and 317 billion USD annually (Rothwell, 2016). Healthy aging and with it the improvement of the quality of life of the elderly population is a major challenge for society. Key aspects of health and the ability to accomplish daily tasks are balance and stability (Alexander, 1994) which have been focus of extensive research. Especially healthy “golden agers”—senior individuals in the age range 55–70—are of interest since, on the one hand, deterioration in balance function accelerates after reaching the approximate age of 60 (Era et al., 2006) and, on the other hand, this age group is usually still active and thus a suitable target for preventive programs. In this context gender effects or interactions between gender and aging on postural control are also interesting, because literature is somewhat incoherent on this issue: there is evidence that the two genders are unequally influenced by aging (Evans and Hurley, 1995) and postural control may be affected differently (Wolfson et al., 1994). Other authors found that some of the gender effects on balance performance disappear when normalizing, for example, to body height (Bryant et al., 2005).

Balancing abilities and the progress of fall prevention programs are assessed with variables that quantify postural control through measures of body sway, for instance the total amount of sway, sway velocity, sway range, amongst others. These variables typically reveal age effects when comparing young healthy to frail old subjects, particularly if the sample size is very large (Era et al., 2006), if task difficulty is increased or instabilities are artificially created (eyes-closed, unstable surfaces, moving surfaces, waist-pulls, etc.; Rogers and Mille, 2003; Cavalheiro et al., 2009; Tardieu et al., 2009), or in dual-task situations with an additional cognitive task (Bernard-Demanze et al., 2009; Manor et al., 2010). Nevertheless, body sway variables are often unable to detect age effects when performing tasks that involve postural control and when comparing healthy young to older, but healthy, non-frail participants (Bernard-Demanze et al., 2009).

The body sway variables are usually derived from center of pressure (COP) trajectories which integrate all of the postural movements of the whole body into one 2-dimensional variable. Hence, to some degree, studies that assess such sway variables and interpret the results on a physiological basis interpret the control system as a whole. Implicitly it is assumed that physiological changes and adaptations that accompany aging influence the postural movements as a whole. This assumption, however, is not obvious when taking recent theories of motor control into consideration.

Currently one of the most successful models for motor control is the optimal feedback control (OFC) theory (Todorov and Jordan, 2002). This theory predicts that when performing a task, the biomechanical degrees of freedom are controlled depending on their relevance to the task. The more relevant a movement dimension is to the task, the tighter it is controlled. Movement control in this context is usually assessed by quantifying movement variability, with low variability indicating tight and high variability suggesting less tight control. This prediction of the OFC is referred to as “minimal intervention principle” (Todorov and Jordan, 2002) and corresponds to earlier concepts in motor control theory such as the uncontrolled manifold hypothesis (Scholz and Schöner, 1999; Latash et al., 2001; Friedman et al., 2009; Arpinar-Avsar et al., 2013; Park and Xu, 2017). However, if different movement dimensions are controlled differently, then one could hypothesize that aging effects may emerge differently in different dimensions or that only specific dimensions, for instance only the most task-relevant ones, reveal aging effects.

The term “movement dimension” relates to the mechanical degrees of freedom within the moving system (Todorov and Jordan, 2002). However, when analyzing whole-body postural control movements, it seems more appropriate to investigate synergistic, functional movement components such as the ankle or hip strategies (Nashner and McCollum, 1985; Woollacott et al., 1986; Winter, 1995; Ting and Macpherson, 2005). Principal component analysis (PCA) has been applied in previous research studies as a method for determining synergistic postural movement components (Troje, 2002; Daffertshofer et al., 2004; Verrel et al., 2009; Federolf et al., 2012a,b, 2013; Federolf, 2016). The movement components resulting from a PCA (called *principal movements* PMs) are one-dimensional whole body movement components that together form the original movement. Applying a PCA allows to assess the coordinative structure on the one hand and the control of individual postural movements on the other. The coordinative structure is represented by the amount of activity of the individual PMs. Differences in the relative contribution of the PMs show that the interplay of PMs and hence the coordinative structure of this movement is different (Zago et al., 2017). The control of individual postural movements can by analyzed using the PM-accelerations. The PM-accelerations are a direct result of the control system, since accelerations are directly proportional to the acting forces, which, in the case of postural control, are directly produced or allowed for by the neuromuscular control system (Federolf, 2016).

The purpose of the current study was to investigate age effects in the postural movements observed in healthy volunteers standing in a tandem stance. Specifically, the hypotheses were tested that (i) age effects would manifest in the control of only specific movement components; that (ii) the characteristics of how age groups control specific movement components would differ between different movement components; and we speculated that (iii) the emergence of aging effects or of how the age groups control specific movement components would be linked to the how task-relevant the movement components are. Furthermore, we tested for gender effects and age-by-gender interactions.

## Methods

### Subjects

The current paper analyzes data from a previous, unpublished study in which 106 subjects had volunteered for several balance and walking measurements. The study was conducted in agreement with the Declaration of Helsinki, particularly, an institutionalized ethics review board had approved the study design and informed written consent was obtained from all volunteers prior to any measurements. The inclusion criteria of the original study were (i) the age of the volunteer ranged either between 20 and 35 (young group) or 55 and 70 (older group); (ii) subjects were in good health with neither cardiovascular nor neurophysiological issues (this criterion was self-reported); (iii) subjects had an occupation that required them to be on their feet (standing, walking) roughly 50% of their time; (iv) we accepted volunteers who conducted occasional recreational sports activities, but no athletes who regularly trained for a specific sport. For the current study, a sub-set of 25 young and 25 older subjects were selected based on the following additional criterion: (v) the volunteers completed one of two trials of the tandem stance without visual instabilities, specifically, without taking a step, removing the hands from the hip, or extensive balance movements. Fulfillment of this criterion was evaluated during post-processing based on the datasets. Three investigators performed the evaluation for criterion (v) independently and the inclusion decision was based on the majority vote. This additional criterion was introduced since the goal of the current study was to compare postural control between age groups unaffected by occurrences of instability events. Of the two resulting test groups, the young group contained 17 women and 8 men aged 25 ± 3 years, height 1.69 ± 0.09 m, and weight 73 ± 18 kg (mean ± standard deviation). The older group also contained 17 women and 8 men, aged 59 ± 4 years, height 1.64 ± 0.09 m and weight 72 ± 12 kg.

### Measurement procedures

The volunteers performed 80-second tandem stances with their feet placed in one line such that the toe of the rear foot barely touched the heel of the front foot. The tandem stance is a task of medium difficulty level that is often used in balance assessments and can be easily performed by both, the older and the younger participants. The tandem stance position is well-suited to test the minimal intervention principle. The participants could test both positions and then choose whether they preferred the right or the left foot in front. The subjects were further instructed to keep their hands on the hip and to “stand as steady as possible.”

**Figure 1:**
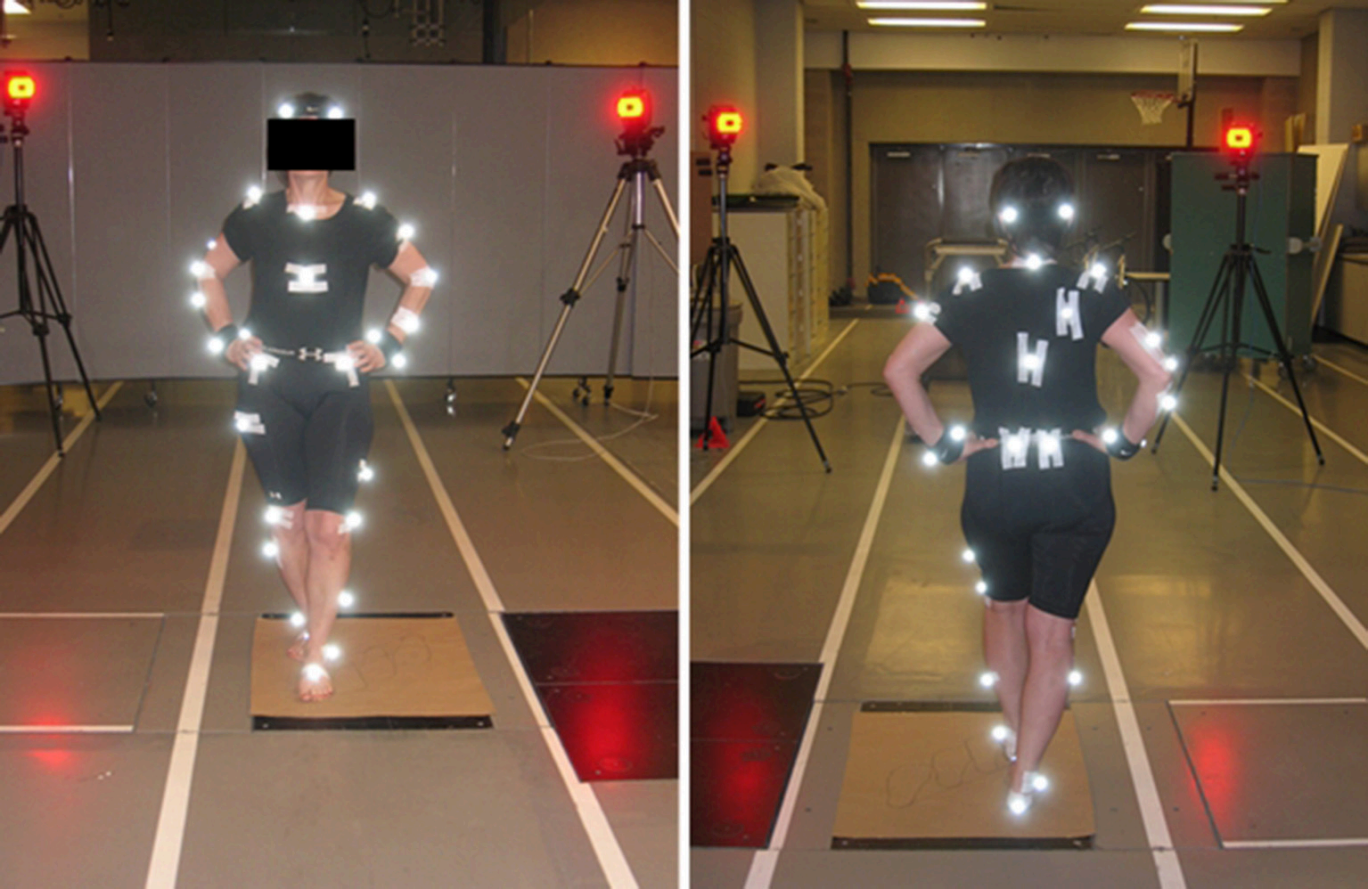
Volunteer equipped with 37 reflective markers performing a tandem stance.

The volunteers were equipped with 37 reflective markers (standard plug-in gait marker set without markers on the hands; see Figure 1), whose positions in space were recorded at 240 frames per second by 8 synchronized video cameras (Motion Analysis Corp., Santa Rosa, CA, USA). The 3D marker trajectories were reconstructed using the software EvaReal-Time (“EvaRT”; MotionAnalysisCorporation, Santa Rosa, CA, USA). Gaps in marker trajectories were filled using a PCA-based reconstruction technique (Gløersen and Federolf, 2016).

### Data analysis—principal component analysis (PCA)

The data analysis procedures were implemented in Matlab® (The MathWorks Inc., Natick, MA, USA). Non-symmetric mid-segment markers were omitted for the analysis. If subjects stood with the left foot in front, then their data were mirrored and relabeled such that all datasets for the analysis described tandem stances with the right foot in front. To address anthropometric differences, the dataset of each subject was centered by subtracting the mean posture and normalized by dividing by the subject's height. Then each marker was weighed with the relative segment mass it represents (Federolf, 2016; Gløersen et al., 2018). Finally, the normalized data of all subjects were concatenated into one input matrix for the PCA.

The underlying idea for the PCA conducted in the current study was that all marker coordinates at a given time are interpreted as a posture vector, i.e., as a position in posture space (Troje, 2002; Daffertshofer et al., 2004). By performing the PCA on the observed posture vectors variables were obtained that quantify postural variability (Troje, 2002; Daffertshofer et al., 2004; Verrel et al., 2009; Bockemühl et al., 2010). Specifically, a PCA yields *eigenvalues* EV_k_*, eigenvectors* PC_k_, and *scores*, where *k* denotes the order of the eigenvector. With the normalization used in the current study, the *eigenvectors* form the basis of a coordinate system in which postural movements can be compared between subjects. For an intuitive interpretation of the PC_k_ it has been suggested to regard the postural movements they resemble as *principal movements* PM_k._ (Federolf et al., 2012a).

The *scores*, which are obtained by projecting the individual normalized posture vectors onto each PC_k_, can be interpreted as the positions in posture space, i.e., as *principal positions* PP_k_(t). The PP_k_(t) are therefore time series that describe the temporal evolution of a specific PM_k._

The *eigenvalues* EV_k_ quantify the contribution of the associated PM_k_ to the overall variance. The EV_k_ were therefore not subject specific in the current study. It has been proposed to calculate *relative variances* rVAR_k_ of the *scores* as subject-specific variables that directly correspond to the EV_k_ and quantify how much each PM_k_ contributed to this subject's overall postural variance (Federolf et al., 2013). However, as both rVARs and EVs quantify the variance in the data, they are proportional to the square of the postural movement amplitude. Therefore, in the current study the square roots of the subject specific variances were calculated to compute *relative standard deviations* rSTD_k_, to obtain variables that scale directly to postural movement amplitudes. For each subject, the rSTD_k_ quantify the percentage of the subject-specific overall postural motion that is explained by each PM_k_. If systematic differences exist in the rSTDs between groups, then this indicates a difference in the coordinative structure of the postural movements in the sense that specific PMs are more or less important for the subjects' overall postural sway.

### Kinematics in posture space and variables characterizing the control of postural movements

Score-time series PP_k_(t) quantify a subject's position in posture space, which is spanned by the orthonormal basis {PC_k_}. Hence, their first derivatives quantify the *principal velocities* PV_k_(t) := d/dt PP_k_(t), and their second derivatives the *principal accelerations* PA_k_(t) := d2/dt2 PP_k_(t) of the postural movements PM_k_ (Federolf, 2016).

**Figure 2:**
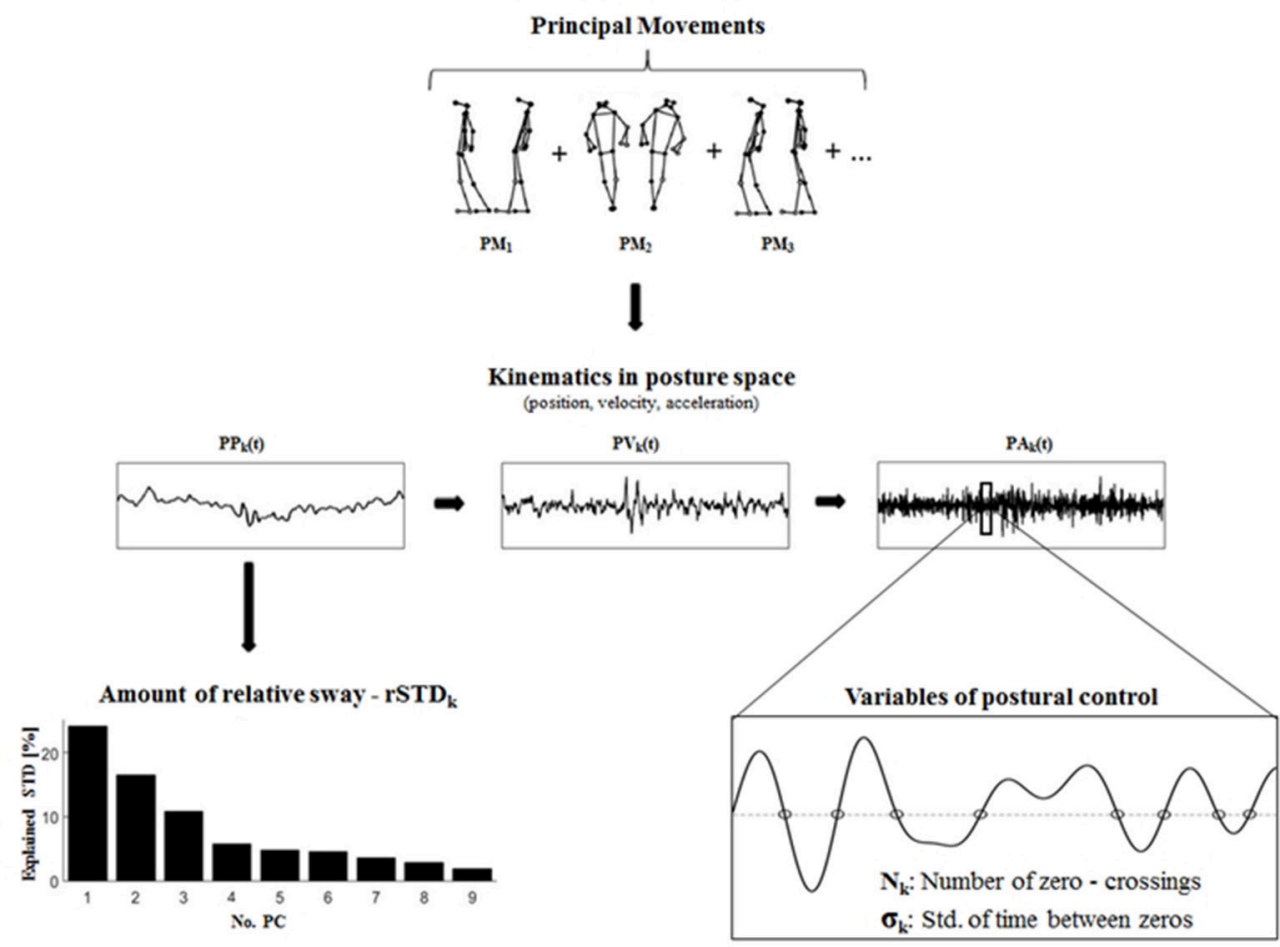
The computation of the variables rSTD_k_, N_k_, and σ_k_ from results of the PCA.

In static balance exercises only two types of forces produce accelerations that cause postural changes: gravity—which is constant—and muscle action. Therefore the PA_k_(t) can be regarded as time-series that quantify the neuro-muscular system's control of the PMs (Federolf, 2016). In the current study two new variables were used to characterize the control of PMs. First, the number of zero-crossings N_k_ of the PA_k_(t) time-series were calculated. The underlying rational was that PA_k_(t) cross zero whenever the direction of the acceleration changes, i.e., when the postural control system counteracts the current postural acceleration. The N_k_ thus serve as a measure for how tightly the neuro-muscular system controls the motion of a postural movement component. Second, the standard deviations of the *time between zero crossings* σ_k_ were calculated in each subject's PA_k_-time series. The σ_k_ might be interpreted as a measure of the regularity or irregularity of the neuromuscular interventions in the control of a PM. A schematic illustration of the data analysis steps is shown in Figure 2.

### Validity considerations and cross-validation

A Fourier analysis was conducted on the PP_k_(t) time series, which revealed that the highest power resided in frequencies around 2–3 Hz, but visible power was still found in the frequency range between 4 to 7 Hz. The PP_k_(t) were therefore filtered with a 5th-order Butterworth lowpass filter using a cut-off frequency of 7 Hz. To calculate the PA_k_(t), a lowpass FIR-differentiator of order 50 was used with a passing frequency of 5 Hz and a stopping frequency of 7 Hz to avoid phase-shifts across frequency bands. Despite the filtering, N_k_ and σ_k_ are both highly susceptible to noise, as they are both calculated from PA_k_(t), a variable obtained through a double differentiation. Therefore, a detailed discussion on the effects of filtering on the magnitude of these variables and on the statistics is included in the Supplementary Materials.

A leave-one-out cross-validation was conducted to evaluate the vulnerability of the PMs and the dependent variables to changes in the input data. The first nine PMs were found to be robust to changes in the input, i.e., the PC-vector did not change its orientation in posture space by more than 15° in the cross-validation analysis. The statistical analysis was therefore limited to the dependent variables derived from these 9 PMs.

### Statistics

All statistics were computed with IBM SPSS Version 24. Kolmogorov-Smirnov and Shapiro–Wilk tests were conducted to test for normality and Levene's tests were applied to assess equality of variances. To test for age and gender effects in the rSTD_k_, N_k_, and σ_k_, two-way ANOVAs and *post-hoc* comparisons with Sidak-correction were computed. The significance level was set to α = 0.05. For each statistically significant result we report the relative change of the mean value (younger or male group = 100%), *p*-value, partial eta squared η_*p*_^2^ and the observed power π. An overview of the results for all variables, groups, and PMs (means ± standard deviation) is included as Supplementary Materials.

## Results

**Figure 3:**
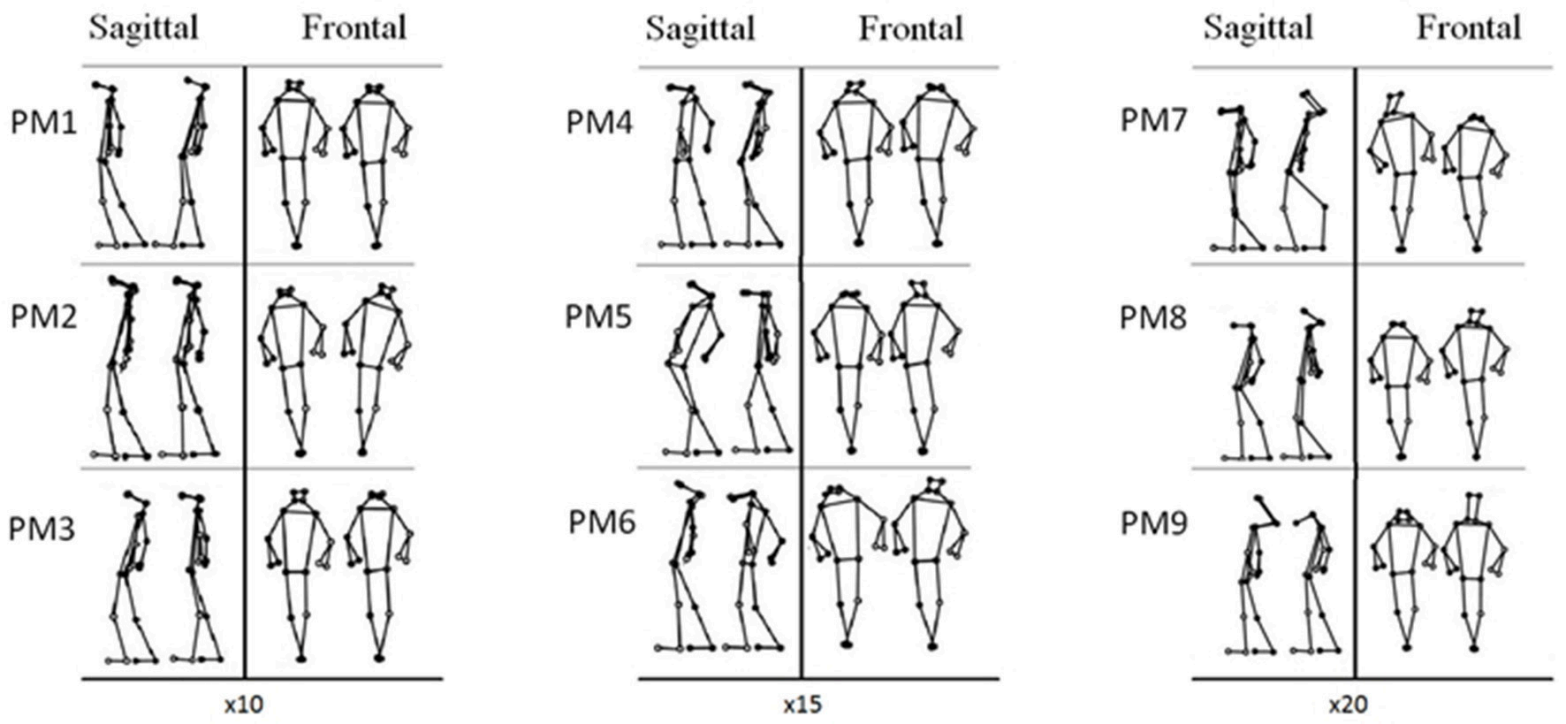
Each graph shows the two opposing extreme positions of the PMs of the first subject of the younger age group in the frontal and sagittal planes. The postural changes were amplified by a factor 10, 15, 20, in the first, second and third column, respectively.

The first nine PMs explained 98.2% of the overall postural variance. The aspects of postural control that each PM resembled are listed in Table 1 and visualized in Figure 3 and the videos submitted as Supplementary Materials.

**Table 1:** Description of principal movement components PM_k_.

**k**	**EV [%]**	**Effects**	**Main strategy (directions)**	**Specifications/additional features**
1	53.11	**–**	Ankle sway (anterior/posterior)	Minor compensatory right knee flexion/extension.
2	25.05	^*^, ^X^	Ankle sway (medial/lateral)	Minor compensation with flexion/extension in both knees.
3	10.67	^X^	Upper body retraction	Upper body leans back. Flexion/extension in right/front knee.
4	3.08	^&^	Upper body rotation (around front leg)	Left hip frontal/dorsal sway (stationary right hip).
5	2.12	**–**	Upper body rotation (around back leg)	Right hip frontal/dorsal sway with right knee flexion/extension.
6	1.87	^&^	Upper body sway (medio/lateral)	Hip is abducted/adducted. Both knees display flexion/extension.
7	1.23	**–**	Knees (flexion/extension)	Opposite flexion/extension and abduction/adductions in the hips.
8	0.76	^*^	Left knee (flexion/extension)	Additional head retraction and upper body elevation.
9	0.31	^*^	Head nodding (protrusion/retraction)	Head is moved from down-front to up-high and back.

The eigenvalues EV_k_ quantify the respective contribution of each PM to the overall variance in percent. Significant age, gender, or interaction effects are symbolized by *, &, X, respectively.

In PM_2_, which predominantly represented medio-lateral ankle sway, significant age effects were found in both the coordinative structure {rSTD_2_: +13.1% [old vs. young], *F*_(1, 46)_ = 6.28, *p* < 0.016, η_*p*_^2^ = 0.12, π = 0.69} and the control variables [N_2_: −9.2%, *F*_(1, 46)_ = 8.05, *p* = 0.007, η_*p*_^2^ = 0.15, π = 0.79; and σ_2_: +13.6%, *F*_(1, 46)_ = 7.4, *p* = 0.009, η_*p*_^2^ = 0.14, π = 0.76]. The control variables showed no age-by-gender interaction, however, rSTD_2_ showed a significant interaction effect [rSTD_2_: *F*_(1, 46)_ = 6.45, *p* = 0.015, η_*p*_^2^ = 0.12, π = 0.70]. The *post-hoc* analysis revealed that the increase with age in rSTD_2_ originated only from the male group, which showed a 46% increase with age (*p* = 0.004, η_*p*_^2^ = 0.17, π = 0.85), while the female group showed no changes (−0.3%, n.s.).

In PM_3_, which coupled an upper body retraction with a flexion of the front knee, no main effects were found. The age-by-gender interaction was significant in rSTD_3_ [*F*_(1, 46)_ = 5.38, *p* = 0.025, η_*p*_^2^ = 0.10, π = 0.62] but the *post-hoc* comparisons between sub-groups were not significant.

Women exhibited a different coordinative structure as they displayed more relative sway in PM_4_ [rSTD_4_: +23.9%, *F*_(1, 46)_ = 4.43, *p* = 0.041, η_*p*_^2^ = 0.09, π = 0.54] and less in PM_6_ [rSTD_6_: −17.8%, *F*_(1, 46)_ = 4.31, *p* = 0.043, η_*p*_^2^ = 0.09, π = 0.53] than men. The variable quantifying the amount of control intervention suggested that women controlled PM_4_ less tightly than men [N_4_: −5.7%, *F*_(1, 46)_ = 4.39, *p* = 0.042, η_*p*_^2^ = 0.09, π = 0.54] but exerted similar control in PM_6_.

The variables that quantify the control of PM_8_ and PM_9_ displayed three age effects [N_8_: +4.7%, *F*_(1, 46)_ = 5.82, *p* = 0.020, η_*p*_^2^ = 0.11, π = 0.66; N_9_: +2.5%, *F*_(1, 46)_ = 4.34, *p* = 0.043, η_*p*_^2^ = 0.09, π = 0.53 and σ_9_: −8.8%, *F*_(1, 46)_ = 5.37, *p* = 0.025, η_*p*_^2^ = 0.10, π = 0.62]. The relative sway did not show age effects in these PMs.

## Discussion

### Main result

The current study investigated age related differences in postural control between young adults and healthy, active golden agers. Based on considerations deducted from the OFC theory (Todorov and Jordan, 2002), it was predicted that (i) age effects would manifest in the control of only specific movement components; that (ii) the characteristics of the control of these movement components may differ between different movement components; and we speculated that (iii) the emergence of aging effects would be linked to the task-relevance of the movement components.

The first two hypotheses were confirmed in the present study: age main effects were observed specifically in the control characteristics of PM_2_, PM_8_, and PM_9_, but not in other movement components. How age affected the control of these movement components differed between PM_2_ compared to PM_8_ and PM_9_: In PM_2_ the younger group exerted a tighter (N_2_ young > N_2_ old) and more regular (σ_2_ young < σ_2_ old) movement control while in PM_8_ and PM_9_ the older group exerted a tighter and more regular (only PM_9_) control. This is a remarkable result since the various sensorimotor and physiological changes that accompany aging are usually considered to affect the whole system (Bernard-Demanze et al., 2009; Jiménez-Jiménez et al., 2011; Papegaaij et al., 2014). Thus, a priori, it seems counterintuitive that aging effects in postural control would emerge only in specific postural movement components and that different components may even exhibit contrasting control characteristics.

The third hypothesis was supported by the findings in PM_1_ and PM_2_. While PM_1_ contributed more to the overall postural variations than PM_2_ (EV_1_ > EV_2_), PM_1_ represented an anteroposterior sway and PM2 a lateral sway. In the tandem stance it seems obvious that PM_2_ is a critical movement to control for maintaining postural stability in both age groups, while the different PM_1_ states are task-redundant. The age effect observed in PM_2_ suggests that the older age groups were not able to control this critical movement component as well as the younger groups. This seems plausible from a physiological point of view: aging is generally associated with deleterious changes of the sensorimotor system (Papegaaij et al., 2014). These changes arise at different levels of the sensorimotor pathway. The muscle fibers' structures degenerate (Hepple, 2003) and motor-units get enlarged (Campbell et al., 1973). Their activation is driven by the nervous system that undergoes significant changes associated with aging as well. Overall, both the motor and the proprioceptive sensory neurons deteriorate (Papegaaij et al., 2014; Vaughan et al., 2017), modifying cortical and subcortical structures, thus leading to a reorganization of the control mechanisms (Papegaaij et al., 2014). It is very likely that the slowing down and reorganization of the information conduction and processing contribute to the findings in PM_2_ of the older age group showing less frequent (N_2_) and more variable (σ_2_) changes in postural accelerations compared to the young volunteers.

A notable finding in the PM_2_ motion was that the increase of rSTD_2_ due to aging originated from the male population only, while the movement control variables N_2_ and σ_2_ changed equally in the two genders. An increase in rSTD_2_ could be a sign that the older men were not able to control the overall PM_2_ sway amplitude. There are indications in the literature that in compromised postural control, e.g. after a concussion, sway amplitudes regenerate faster, while differences in how postural movements are controlled are detectable for a longer period (Cavanaugh et al., 2006). The current study's finding might indicate the inverse process: aging seemed to have led to changes in the control characteristics, however, it might be speculated that the female participants were still able to control the sway amplitudes similarly to their younger counterparts, while in the male group an effect on the sway amplitude was already detectable. In general, the overall body sway increases as a function of height (Gage et al., 2004). Nevertheless, we expect that height differences can be excluded, since the data was normalized to height. Since men show more relative sway in the dimension that is harder to control, it is possible that woman suffer less from aging impairments than men. It has been shown that strength levels in woman decline at slower rates (Evans and Hurley, 1995). Speculatively, it might be harder for men to adapt to the loss of strength, which could also affect balance capabilities.

The age effects found in N_8_, N_9_, and σ_9_ suggested tighter and more regular control of the PM_8_ and PM_9_ components in the older group compared to the younger group. The reason why age effects were only evident in the control but not the relative sway variables of these components might be due to the fact that both components contributed <1% to the overall variance. Variability within the subjects might be of the same magnitude as the contribution itself, causing differences to remain undetected. Nevertheless, if interpreted within the paradigm of the OFC, then this finding suggests that for the older group the control of these two movement components is more task-relevant than for the younger group. PM_8_ and PM_9_ are both characterized by large head movements. Several previous studies found that the postural control in older volunteers relied more on visual information compared to younger subjects (Woollacott et al., 1986; Prioli et al., 2005). Since head movements could affect the visual cuing it seems plausible that older volunteers need to control head movements more tightly. In fact, aging effects in the head-trunk coordination have already been reported in standing (Paquette et al., 2006) and in walking (Kavanagh et al., 2005; Paquette et al., 2006).

### Gender effects

Hip flexion and upper body rotations (PM_4_) contributed more to the overall postural movements and seemed less tightly controlled in the women groups compared to the men groups, while upper body medio-lateral sway and hip ab-adduction movements (PM_6_) contributed less. These effects must be interpreted with caution since the effect in N_4_ is only significant for the chosen filter (see “FilteringSelection_Statistics.docx,” section 2. Gender comparisons). Nevertheless, differences were observed despite normalizing the current data to body height, contrary to previous research suggesting that gender differences in total sway disappear when normalizing to body height (Bryant et al., 2005). The anatomical differences or differences in relative strength (Claiborne et al., 2006) between men and women are the most likely explanations for these differences in the movement structure. Another possibility is that men and woman relied on slightly different hip strategies while performing the tandem stance.

### Methodological aspects of the current paper

The current study built on the concepts from the OFC theory (Todorov and Jordan, 2002) and the uncontrolled manifold hypothesis (Scholz and Schöner, 1999; Latash et al., 2001; Friedman et al., 2009; Arpinar-Avsar et al., 2013; Park and Xu, 2017), however, contrary to these concepts, the approach taken in the current study is fully data-driven and does not require the a priori postulation of a cost function or a task variable. Further, the current study is also based on the concept of kinematics in posture space, a novel concept that has recently been validated biomechanically by demonstrating that the PP_k_(t) and PA_k_(t) predict the center of pressure COP trajectory (Federolf, 2016). The two new variables N_k_ and σ_k_ introduced in the current study offer an alternative approach to characterizing how tightly and how regularly a movement component is controlled by the neuro-muscular system, while most previous studies characterized these aspects of motor control through an analysis of the observed variability in repeated tasks (Friedman et al., 2009; Verrel et al., 2009; Bockemühl et al., 2010; Kobayashi et al., 2014) or use more complex, non-linear variables, such as entropy (Cavanaugh et al., 2006; Manor et al., 2010; Zhou et al., 2015), to characterize regularity.

### Limitations

Although inclusion criterion (v) was assessed by three investigators independently, this criterion for subject selection remains subjective. Furthermore, the number of male volunteers was considerably smaller than the number of female participants, leading to unevenly distributed gender groups.

PCA is a linear decomposition method. The PMs should be seen as a coordinate system for postural movements. Interpreting individual PMs independently from another should be done with caution, since any original postural movement is always a combination of all PMs.

The N_k_ and σ_k_ were computed on acceleration data obtained from dual differentiation of kinematic data. Therefore, the filtering of the data influences the nominal values for N_k_ and σ_k_. This limitation was addressed by repeating the whole analysis with a wide range of filter settings. The results of this additional analysis are attached as Supplementary Data File “FilteringSelection _Statistics.docx.”

## Conclusions and implications

The current study supports the hypothesis that aging effects in postural control emerge in specific movement components, not in all postural movements. This finding should be considered when designing balance training programs for fall prevention or tests for monitoring the progress of participants in such programs.

## Author contributions

TH processed the data and wrote the paper; A-CD provided valuable insights for the discussion and reviewed the paper; BN was the leader of the data collection and approved the manuscript; PF was involved in the data collection. He supervised the data processing and assisted the writing process.

### Conflict of interest statement

The authors declare that the research was conducted in the absence of any commercial or financial relationships that could be construed as a potential conflict of interest. The reviewer BS declared a past co-authorship with the author BN to the handling Editor.

## Supplementary material

Video S1 Visualization of PM_1_–PM_3_.

Video S2 Visualization of PM_4_–PM_6_.

Video S3 Visualization of PM_7_–PM_9_.

Video S4 Exemplary realization of PM_1_–PM_3_ of the first subject of the younger age group.

Video S5 Exemplary realization of PM_4_–PM_6_ of the first subject of the younger age group.

Video S6 Exemplary realization of PM_7_–PM_9_ of the first subject of the younger age group.
