# Supplementary material for: Age Effects in Postural Control Analyzed via a Principal Component Analysis of Kinematic Data and Interpreted in Relation to Predictions of the Optimal Feedback Control Theory
Source: Front Aging Neurosci. 2018 Feb 5;10:22. doi: 10.3389/fnagi.2018.00022 (PMC5807376; doi:10.3389/fnagi.2018.00022)
Supplement: Supplementary file 7 [file DataSheet1.DOCX]

**Supplementary Tables**

The three tables contain the variable’s statistics (mean ± std over the 50 subjects) of the absolute values of the variables relative standard deviation rSTD [%], number of zero-crossings N [#] and standard deviation of time between zero-crossings σ [ms].

|  | **rSTD [%]** | | | |
| --- | --- | --- | --- | --- |
| **PM** | **Male** | **Female** | **Young** | **Old** |
| **1** | 22,5 (+/-8,0) | 23,0 (+/-6,4) | 22,5 (+/-6,1) | 23,1 (+/-7,9) |
| **2** | 20,4 (+/-5,0) | 19,1 (+/-5,5) | 18,3 (+/-5,2) | 20,7 (+/-5,3) |
| **3** | 8,9 (+/-3,1) | 8,6 (+/-3,6) | 8,6 (+/-2,8) | 8,9 (+/-4,0) |
| **4** | 5,1 (+/-1,8) | 6,4 (+/-2,0) | 6,1 (+/-2,0) | 5,9 (+/-2,1) |
| **5** | 4,8 (+/-1,5) | 5,0 (+/-1,7) | 5,4 (+/-1,6) | 4,6 (+/-1,6) |
| **6** | 5,2 (+/-1,7) | 4,3 (+/-1,4) | 4,6 (+/-1,0) | 4,6 (+/-1,9) |
| **7** | 3,6 (+/-1,1) | 3,7 (+/-1,4) | 3,8 (+/-1,3) | 3,6 (+/-1,3) |
| **8** | 2,5 (+/-0,9) | 3,1 (+/-1,1) | 3,1 (+/-1,1) | 2,8 (+/-1,0) |
| **9** | 1,7 (+/-1,0) | 1,6 (+/-0,5) | 1,7 (+/-0,6) | 1,5 (+/-0,8) |

|  | **N [#]** | | | |
| --- | --- | --- | --- | --- |
| **PM** | **Male** | **Female** | **Young** | **Old** |
| **1** | 710 (+/-40) | 682 (+/-55) | 695 (+/-40) | 687 (+/-62) |
| **2** | 565 (+/-77) | 529 (+/-59) | 567 (+/-56) | 515 (+/-67) |
| **3** | 652 (+/-49) | 665 (+/-46) | 652 (+/-46) | 670 (+/-47) |
| **4** | 677 (+/-48) | 639 (+/-67) | 642 (+/-63) | 660 (+/-64) |
| **5** | 746 (+/-42) | 721 (+/-47) | 725 (+/-42) | 732 (+/-51) |
| **6** | 698 (+/-60) | 679 (+/-57) | 677 (+/-54) | 693 (+/-63) |
| **7** | 723 (+/-28) | 729 (+/-53) | 718 (+/-42) | 735 (+/-49) |
| **8** | 782 (+/-45) | 786 (+/-50) | 766 (+/-43) | 802 (+/-47) |
| **9** | 794 (+/-28) | 795 (+/-33) | 784 (+/-32) | 804 (+/-29) |

|  | **σ [ms]** | | | |
| --- | --- | --- | --- | --- |
| **PM** | **Male** | **Female** | **Young** | **Old** |
| **1** | 77 (+/-14) | 81 (+/-17) | 77 (+/-13) | 82 (+/-19) |
| **2** | 121 (+/-23) | 129 (+/-20) | 118 (+/-18) | 134 (+/-21) |
| **3** | 70 (+/-8) | 70 (+/-9) | 71 (+/-8) | 69 (+/-9) |
| **4** | 70 (+/-11) | 77 (+/-14) | 77 (+/-13) | 73 (+/-14) |
| **5** | 55 (+/-7) | 58 (+/-10) | 58 (+/-8) | 56 (+/-10) |
| **6** | 64 (+/-11) | 65 (+/-10) | 66 (+/-10) | 63 (+/-10) |
| **7** | 51 (+/-5) | 50 (+/-8) | 52 (+/-6) | 49 (+/-8) |
| **8** | 43 (+/-4) | 43 (+/-6) | 44 (+/-5) | 42 (+/-6) |
| **9** | 42 (+/-5) | 42 (+/-6) | 44 (+/-6) | 40 (+/-5) |
