# Supplementary material for: Age Effects in Postural Control Analyzed via a Principal Component Analysis of Kinematic Data and Interpreted in Relation to Predictions of the Optimal Feedback Control Theory
Source: Front Aging Neurosci. 2018 Feb 5;10:22. doi: 10.3389/fnagi.2018.00022 (PMC5807376; doi:10.3389/fnagi.2018.00022)
Supplement: Supplementary file 8 [file DataSheet2.DOCX]

**Effect of filtering on the statistics**

The variable rSTD is computed on the PP_k_(t) time-series and changes only slightly when modifying the filtering type and cut-off frequency. This paper makes use of newly developed variables on the PA time-series: the number of zero-crossing N [#] and the standard deviation of the time between zero-crossings σ [ms]. The PA_k_(t) were computed by differentiating the PP_k_(t) twice, resulting in noise amplification.

Since we expect the physiological events occur in the frequency range of 0-7 Hz, the PP_k_(t) were filtered with a 7Hz cut-off low-pass Butterworth filter. The PA_k_(t) were obtained by applying a FIR-differentiator with a stopping frequency of 7 Hz on the PP_k_(t) (see manuscript section 2.5). Nevertheless, the filter choice affects the magnitude of the two variables questioning the reliability of the statistical results.

A lower cut-off frequency will smoothen a PA-signal and result in less number of zero-crossings N. The variable σ can increase or decrease, depending on the signal and the noise. Nevertheless, we would in general expect a lower cut-off frequency to increase this number, because a smoother signal allows larger time intervals and therefore greater differences in the duration of the intervals, hence increasing σ.

Despite the susceptibility of the variables to noise and the filtering choice, we believe that the variables are meaningful and that statistical differences can be assessed in a reliable way, because such effects would influence the magnitude of all variables simultaneously. To support this claim we computed the statistics for various cut-off/stopping frequencies (1-15 Hz in steps of 1 Hz). The PP_k_(t) were filtered with a 5^th^ order Butterworth filter with corresponding cut-off frequency. Then, for all frequencies, the PA_k_(t) were computed and compared in two ways. First, by applying an FIR differentiator with specified stopping frequency (denoted as “FIR” in the graphs) and, second, by twice differentiating the filtered PP_k_(t) and again filtering the results with a 5^th^ order Butterworth (denoted as “Butterworth” in the graphs). This ensured that the filter type did not significantly influence the statistical results. All graphs show an additional red line indicating the significance threshold of p = 0.05.

**Content:**

**1. Age comparisons**

1.1 Age - rSTD

1.2 Age - N

1.3 Age - σ

**2. Gender comparisons**

2.1 Gender - rSTD

2.2 Gender - N

3.3 Gender - σ

**3. Interaction effects**

3.1 Interaction - rSTD

3.2 Interaction - N

3.3 Interaction - σ

## 1. Age comparisons

The variable rSTD does not change notably when changing filter type or cut-off frequency. The age effects remain unchanged for various cut-off frequencies. The main age effects in N_2_ are visible in all cut-off frequencies above 5 Hz. We believe that filtering with a lower frequency the events are filtered out. The other two age effects in N_8_ and N_9_ appear only on a window around 6-10 Hz. Again, a lower filtering cut-off frequency could filter out the effects. We believe in higher frequency ranges noise could affect the statistical results since PM_8_ and PM_9_ are of low amplitude and also N is of smaller magnitude. Similar results can be observed for σ.

**
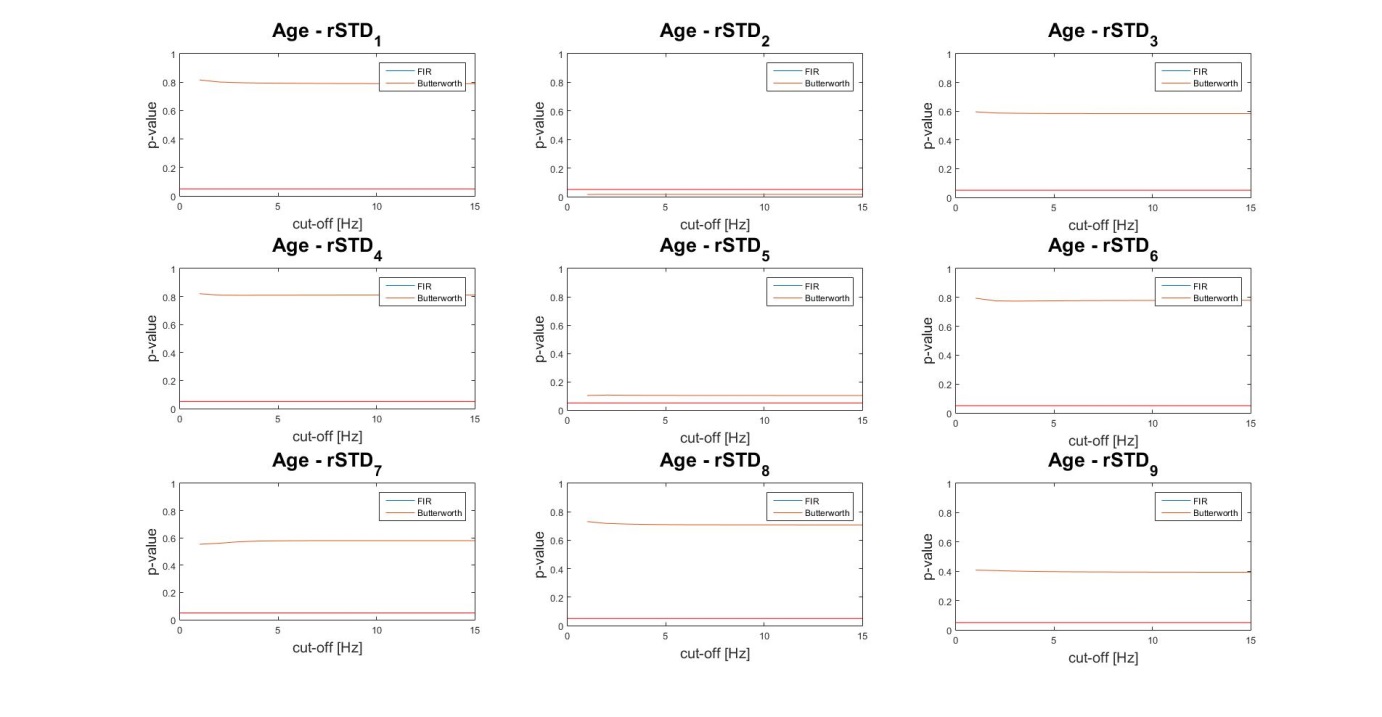
**1.1 Age - rSTD

1.2 Age - N
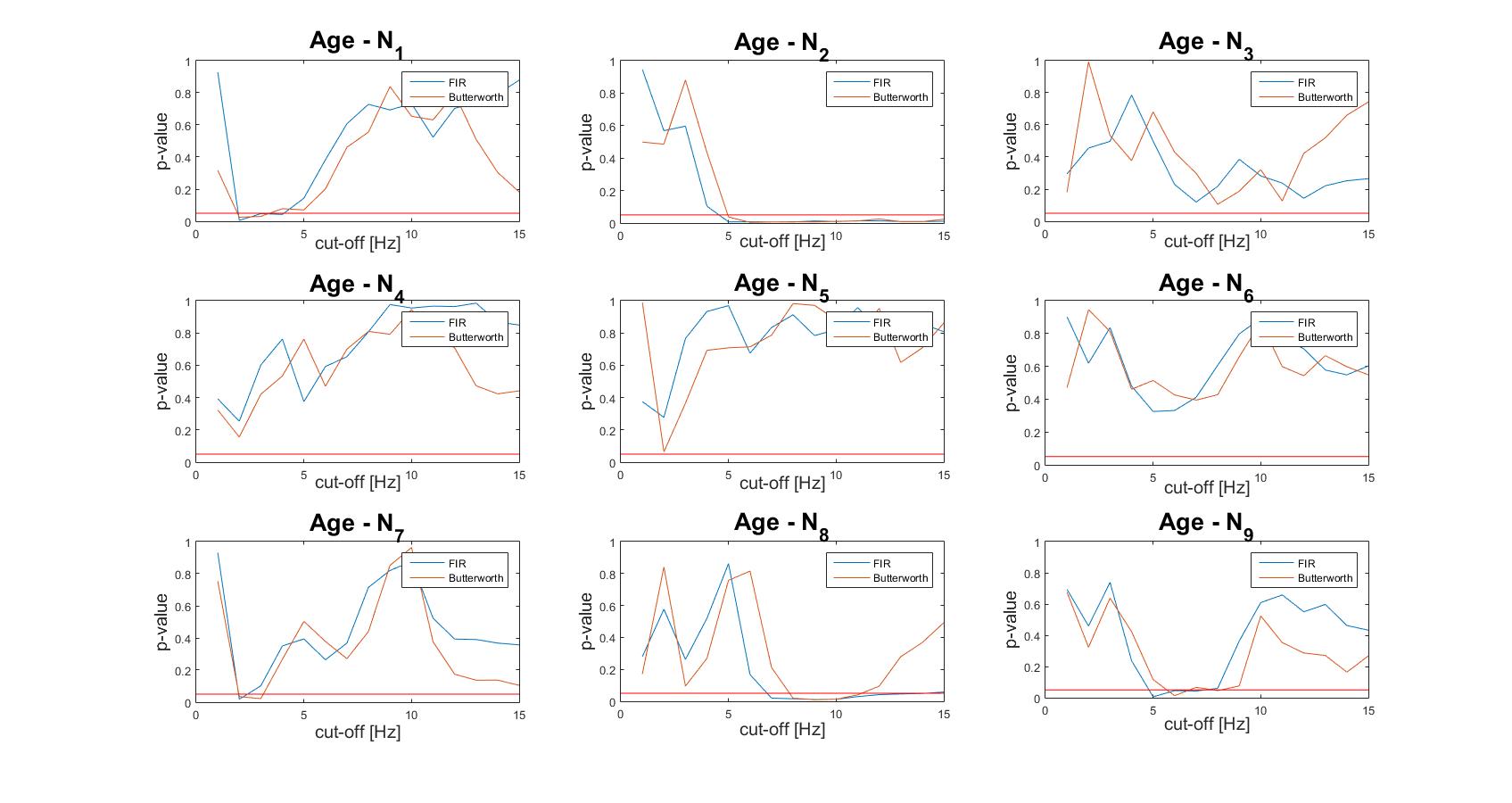


1.3 Age - σ

**
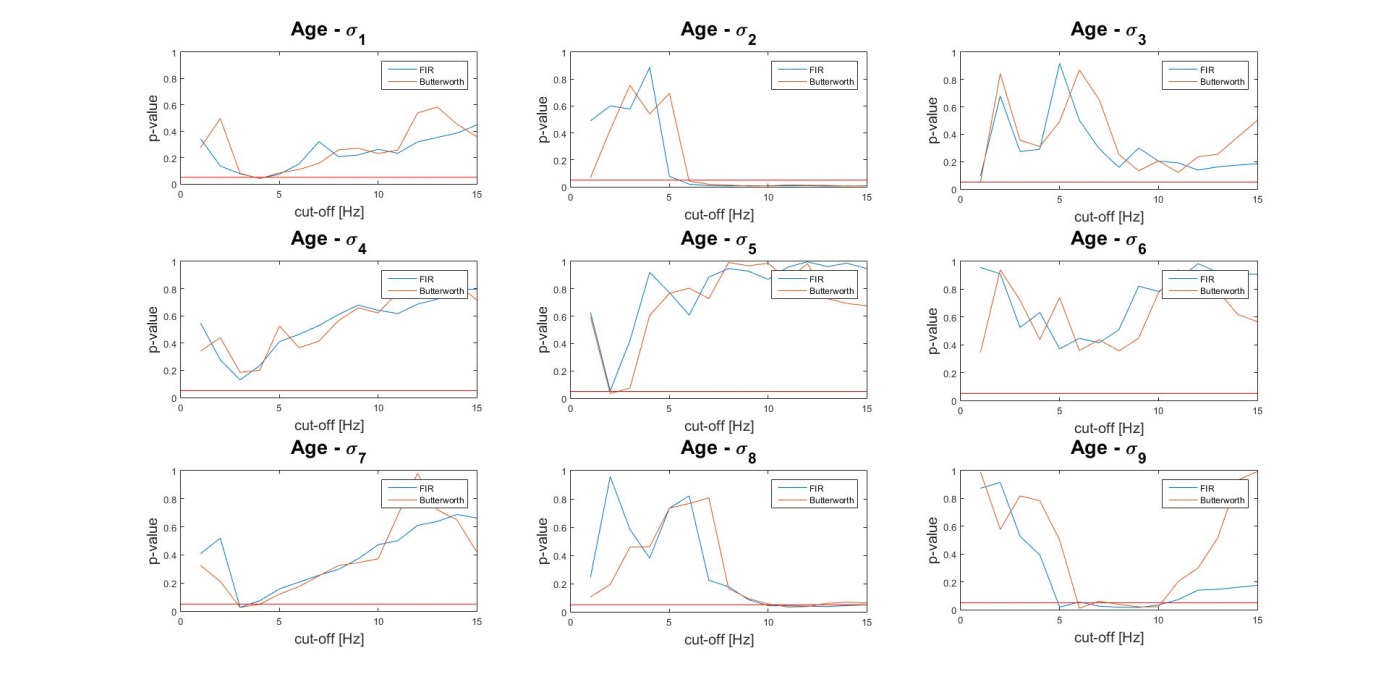
**

## 2. Gender comparisons

The variable rSTD does not change notably when changing filter type or cut-off frequency. The main gender effects in rSTD_4_ and rSTD_6_ remain unchanged. The gender effects in N_4_ are visible only with the chosen cut-off frequency of 7 Hz and the chosen FIR filter (not for the Butterworth filter). The p-values only remain close to significance and might indicate a trend, similar to the p-values of N_5_ and N_1_. N_1_ even reaches significance for higher frequencies but was discarded due to the a priori filter selection. These gender effects for N must therefore be interpreted with caution, as they might also be pure coincidence. Significant gender effects were only found for σ for specific filter choices.


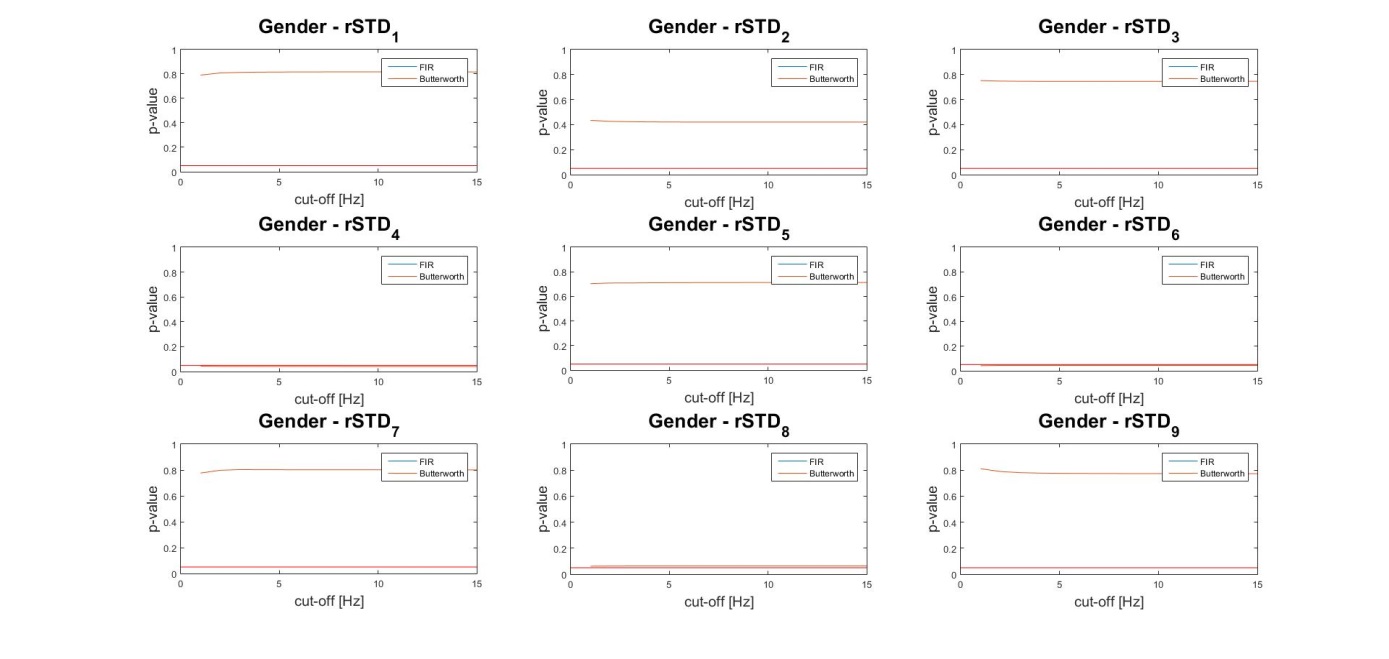
2.1 Gender - rSTD


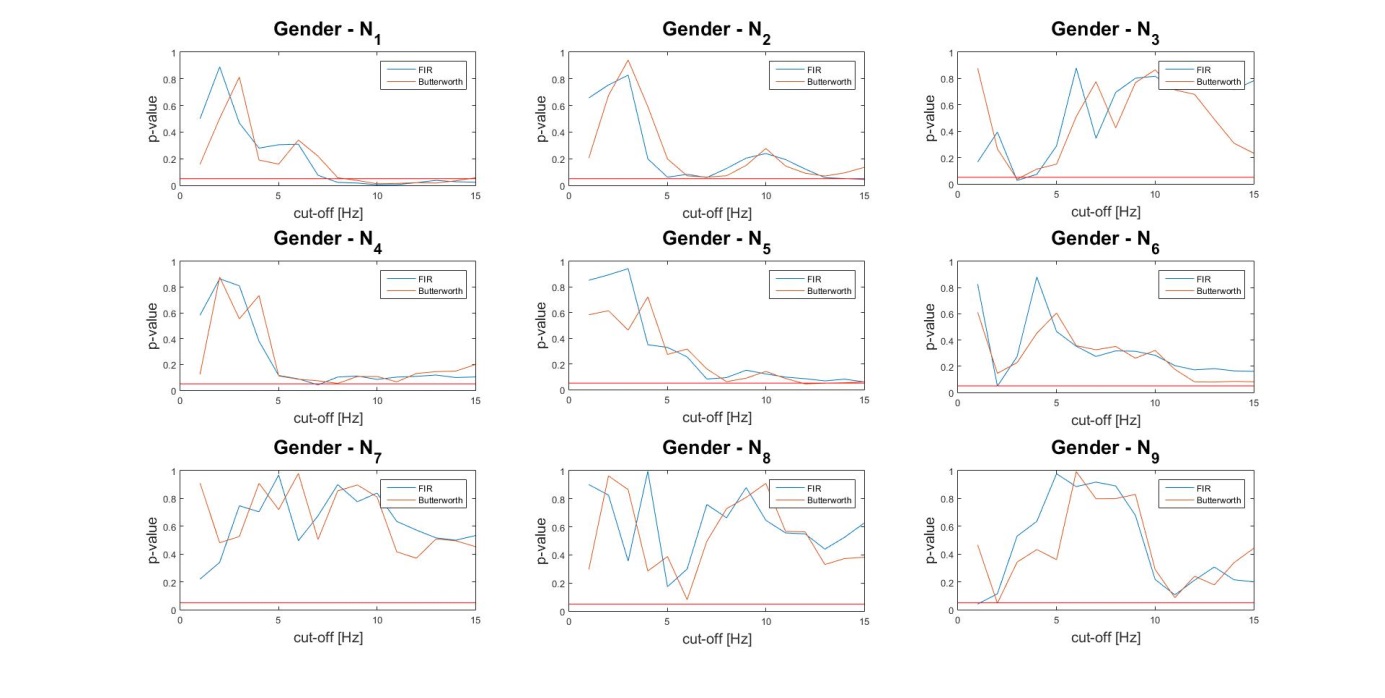
2.2 Gender - N


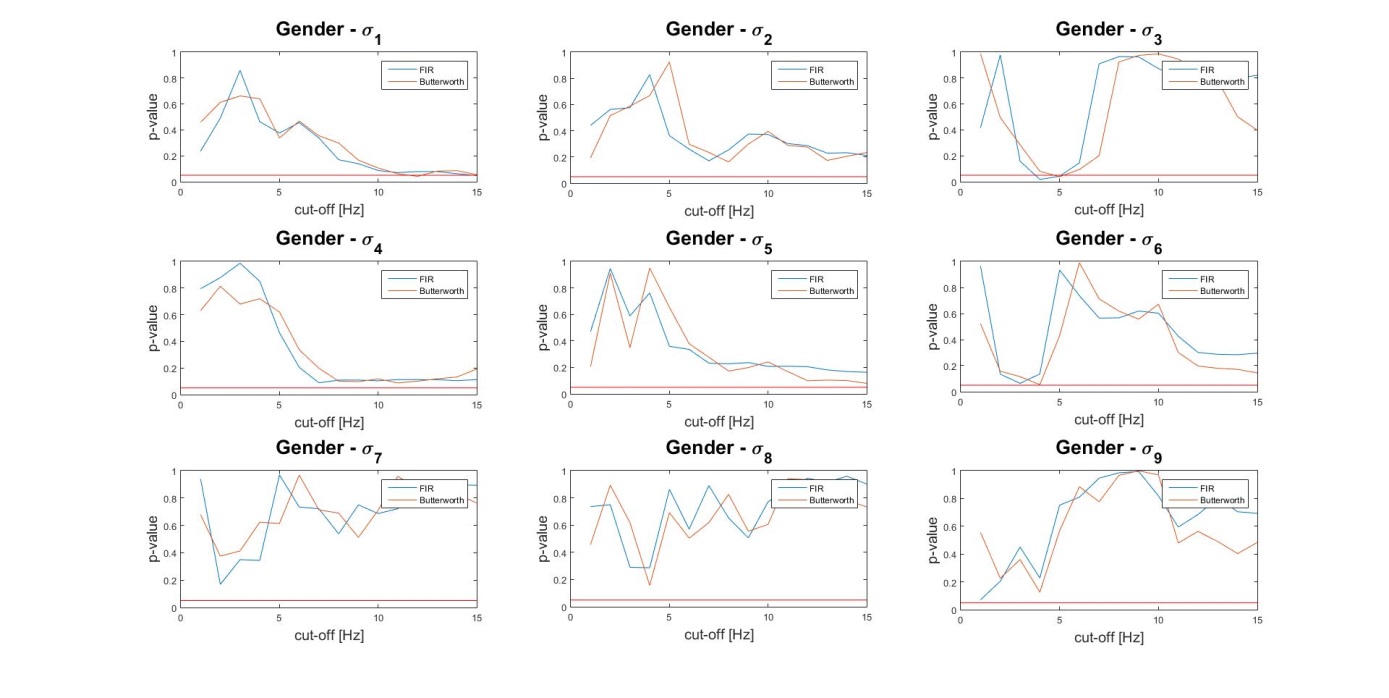
3.3 Gender - σ

## 3. Interaction effects

The two interaction effects in rSTD_2_ and rSTD_3_ are visible for all cut-off frequencies. No other interaction effects exhibit significant p-values, with the exception of specific filter choices.

3.1 Interaction - rSTD


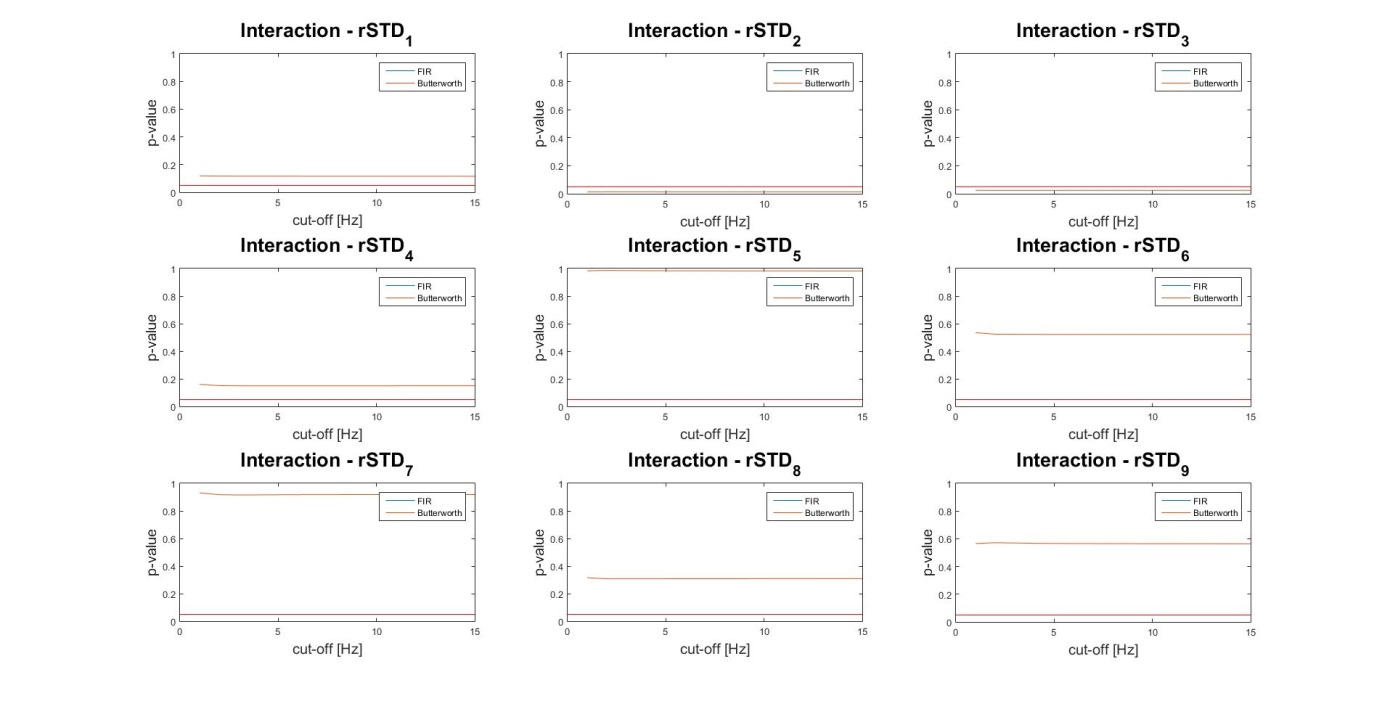


3.2 Interaction - N


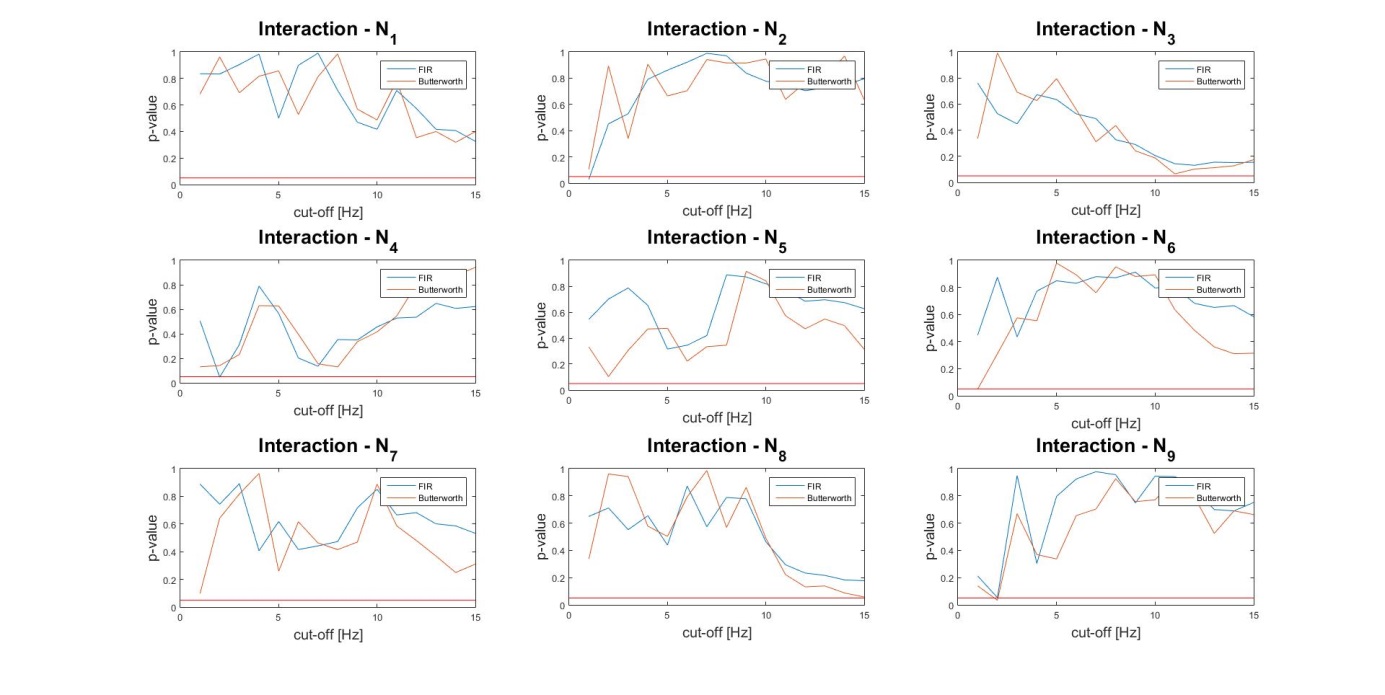


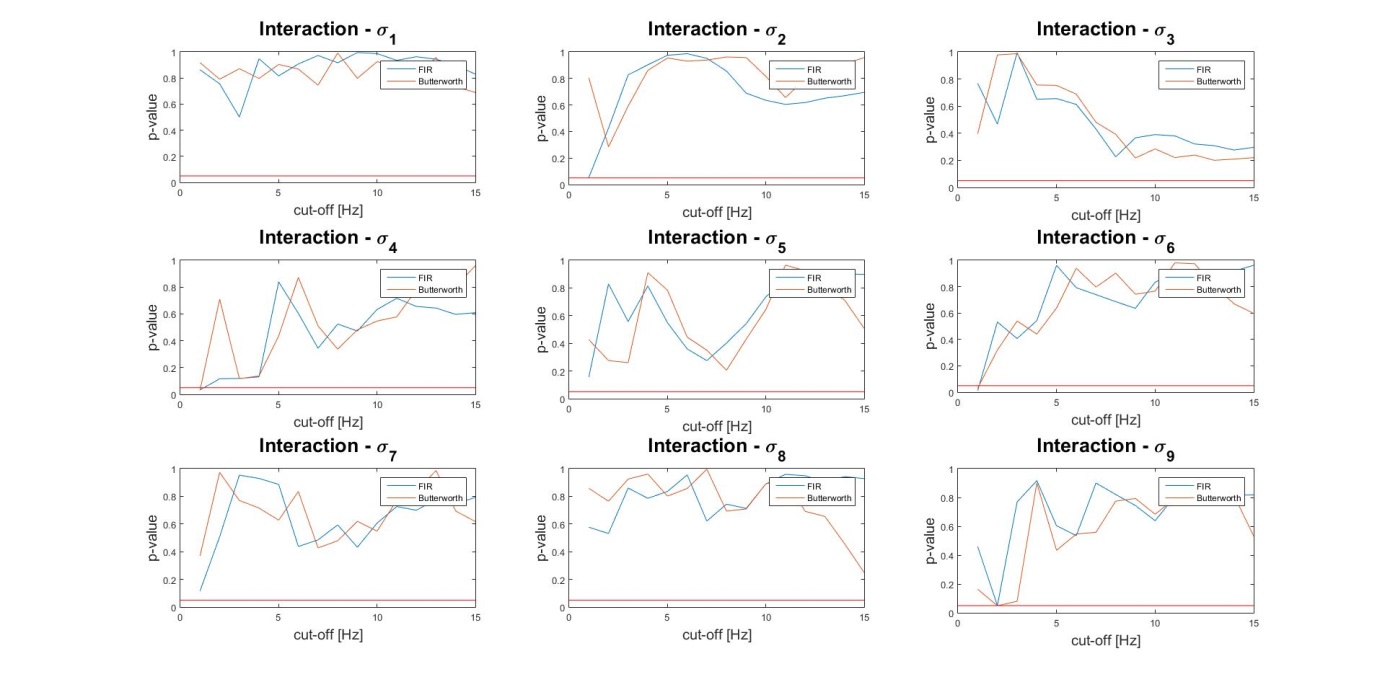
3.3 Interaction - σ
